# Origin of cancer-associated fibroblasts and tumor-associated macrophages in humans after sex-mismatched bone marrow transplantation

**DOI:** 10.1038/s42003-018-0137-0

**Published:** 2018-09-03

**Authors:** Masako Kurashige, Masaharu Kohara, Kenji Ohshima, Shinichiro Tahara, Yumiko Hori, Satoshi Nojima, Naoki Wada, Jun-ichiro Ikeda, Koichi Miyamura, Masafumi Ito, Eiichi Morii

**Affiliations:** 1 Department of Pathology, Graduate School of Medicine, Osaka University, Yamada-Oka, Suita, Osaka 565-0871 Japan; 2 Department of Hematology, Japanese Red Cross Nagoya First Hospital, 3-35 Michishita-cho, Nakamura-ku, Nagoya, Japan; 3 Department of Pathology, Japanese Red Cross Nagoya First Hospital, 3-35 Michishita-cho, Nakamura-ku, Nagoya, Japan

## Abstract

Cancer-associated fibroblasts (CAFs) and tumor-associated macrophages (TAMs) in tumor stroma play a key role in disease progression. Recent studies using mice models suggest that CAFs are partly derived from bone marrow and TAMs primarily originate from bone marrow-derived inflammatory monocytes. However, the origin of these cells in humans remains unclear. Hence, we investigated their human origin, using specimens from human secondary tumors that developed after sex-mismatched bone marrow transplantation, by modified immunofluorescent in situ hybridization analysis and triple immunostaining. We observed that most of the α-smooth muscle actin (αSMA)-positive CAFs in the mammary gland, liver, and oral mucosa specimens obtained 3–19 years after bone marrow transplantation are recipient-derived cells. In contrast, the majority of the peritumoral αSMA-negative fibroblast-like cells are actually bone marrow-derived HLA-DR-positive myeloid cells, such as macrophages and dendritic cells. Furthermore, almost all CD163-positive TAMs and macrophages present in the non-tumor areas are derived from bone marrow.

## Abstract (web summary)

Masako Kurashige et al. investigate the origin of cancer-associated fibroblasts and tumor-associated macrophages in humans. They find that these are derived from bone marrow following sex-mismatched bone marrow transplants.

## Introduction

Tumor stroma has been shown to play a key role in promoting cancer progression and metastasis. This surrounding tissue consists of a variety of cell types implicated in disease progression, including peritumoral spindle-shaped cells, commonly referred to as cancer-associated fibroblasts (CAFs), as well as tumor-associated macrophages (TAMs)^1^. TAMs have been shown to regulate tumor progression via complex interactions with both cancer and stroma cells, including CAFs. Activation of these CAFs via crosstalk with cancer, immune, and other stromal cells results in the expression of α-smooth muscle actin (αSMA), similar to myofibroblasts.

Previous studies have identified CAFs originating from a variety of different cells types, including fibroblasts, endothelial cells, and vascular mural cells^1–3^. Recently, multiple independent studies have also implicated bone marrow-derived cells, most likely mesenchymal stem cells, as a considerable source of CAFs^4–13^. Quante et al.^10^ showed that bone marrow-derived mesenchymal stem cells are actively recruited to the dysplastic stomach, comprising at least 20–25% of αSMA(+) CAFs in a mouse model of inflammation-dependent gastric dysplasia. In contrast, Arina et al.^14^ recently showed that CAFs were derived primarily from local fibroblasts, not from cells in the bone marrow, casting doubt on the origins of these cells.

To our knowledge, there has been only one human study indicating the presence of bone marrow-derived CAFs in histopathological specimens^9^, performed using a combination of Y-chromosome chromogenic in situ hybridization and immunohistochemistry. In that study, bone marrow-derived αSMA(+) CAFs were observed in a gastric carcinoma of a female patient who had received a sex-mismatched allogeneic stem cell transplantation. However, as mentioned by the authors, it was unclear whether the donor-derived cells differentiated into αSMA(+) CAFs or simply fused with already existing αSMA(+) myofibroblasts. If the mechanism by which CAFs developed from donor cells was not cell fusion, their result suggests that, in human hematopoietic stem cell transplantation, bone marrow-derived mesenchymal cells were transplantable and recruited into the tumor site.

Macrophages originate from bone marrow-derived monocytes or yolk-sac progenitors. In mice, tissue macrophages are generally derived from the yolk sac, and in many organs under steady state conditions, they are maintained for >1 year by self-renewal^15–21^. In some organs such as the intestine or depending on the tissue environment, they are replaced by bone marrow-derived cells after the onset of hematopoiesis in the bone marrow^22–25^. TAMs are reported to be derived from bone marrow^26–28^.

In humans, the origins of these cells are poorly understood. In cases of bone marrow transplantation, alveolar macrophages, Kupffer cells, and dermal dendritic cells are completely replaced by bone marrow-derived cells within 1 year following transplantation, while some dermal macrophages remain^29–31^. In contrast, in cases of organ transplantation, various proportions of donor-derived macrophages remain 2–3 years after transplantation^32,33^. There are few reports on the origin of human TAMs; the only exception is a study of lymphoma^34^.

Here we examine the contribution and composition of bone marrow-derived cells to CAF and macrophage populations in clinical specimens of various human carcinomas. For secondary tumors developing after sex-mismatched allogenic bone marrow transplantation, we perform immunofluorescent in situ hybridization (immunoFISH) analysis to simultaneously detect both immunophenotypic markers and sex chromosomes (X and Y) on a single paraffin section. Three-dimensional reconstruction of cells by confocal laser scanning microscopy more precisely reveal the origin of CAFs and macrophages within these samples (mammary gland, liver, and oral mucosa). Moreover, we determine whether cell fusion occurs by counting the number of sex chromosomes in a single CAF nucleus. Few αSMA(+) CAFs are derived from donors, and donor-derived stromal cells are almost absent from the bone marrow. Many αSMA(−) spindle cells, which resemble CAFs morphologically, are bone marrow-derived human leukocyte antigen–antigen D related-positive (HLA-DR(+)) spindle cells, most of which are macrophages or dendritic cells. Our results suggest that cell fusion does not contribute considerably (<1%) to the generation of these cells. The vast majority of CD163(+) macrophages, including tissue macrophages in normal tissue and TAMs, are derived from bone marrow.

## Results

### Sampling

From 2010 to 2014, six patients were diagnosed with secondary tumors after sex-mismatched hematopoietic stem cell transplantation (Table 1). All patients reported no relapse of the primary disease. The period from hematopoietic stem cell transplantation to diagnosis of secondary tumor was 2.4–19.8 years. Pathological diagnoses of the tumors were as follows: invasive ductal carcinoma (breast), hepatocellular carcinoma (liver), squamous cell carcinoma (oral mucosa), adenocarcinoma (stomach), adenocarcinoma (colon), and adenoma with high-grade dysplasia (colon); the former three cases were invasive, and the latter three were non-invasive.

**Table 1:** Summary of patients and secondary tumor characteristics

Case no.	Recipient sex	Age at diagnosis (years)	Primary disease	Type of HSCT	Interval between transplant and diagnosis (years)	Site of secondary tumor	Pathological diagnosis	UICC TNM Seventh
1	F	37	AML	ur-BMT	19.8	Breast	Invasive ductal carcinoma	T2N1M0
2	M	64	CML	ur-BMT	18.5	Liver	Hepatocellular carcinoma	T1N0M0
3	M	66	MDS	ur-BMT	3.6	Oral mucosa	Squamous cell carcinoma	T1N0M0
4	M	67	MPN/MDS	ur-BMT	3.9	Stomach	Adenocarcinoma	TisN0M0
5	M	68	MCL	ur-BMT	7.8	Colon	Adenocarcinoma	TisN0M0
6	F	58	Ph+ ALL	ur-BMT	2.4	Colon	Adenoma with high-grade dysplasia	Not available

*F* female, *M* male, *AML* acute myeloid leukemia, *CML* chronic myelogenous leukemia, *MDS* myelodysplastic syndromes, *MPN/MDS* myelodysplastic/myeloproliferative neoplasms, *MCL* mantle cell lymphoma, *Ph+ ALL* Philadelphia chromosome-positive acute lymphoblastic leukemia, *HSCT* hematopoietic stem cell transplant, *ur-BMT* bone marrow transplants from unrelated donors, *UICC TNM Seventh* UICC TNM Classification of Malignant Tumors, Seventh Edition

### Evidence of donor-derived stromal cells with spindle-shaped nuclei in invasive carcinoma

**Fig. 1:**
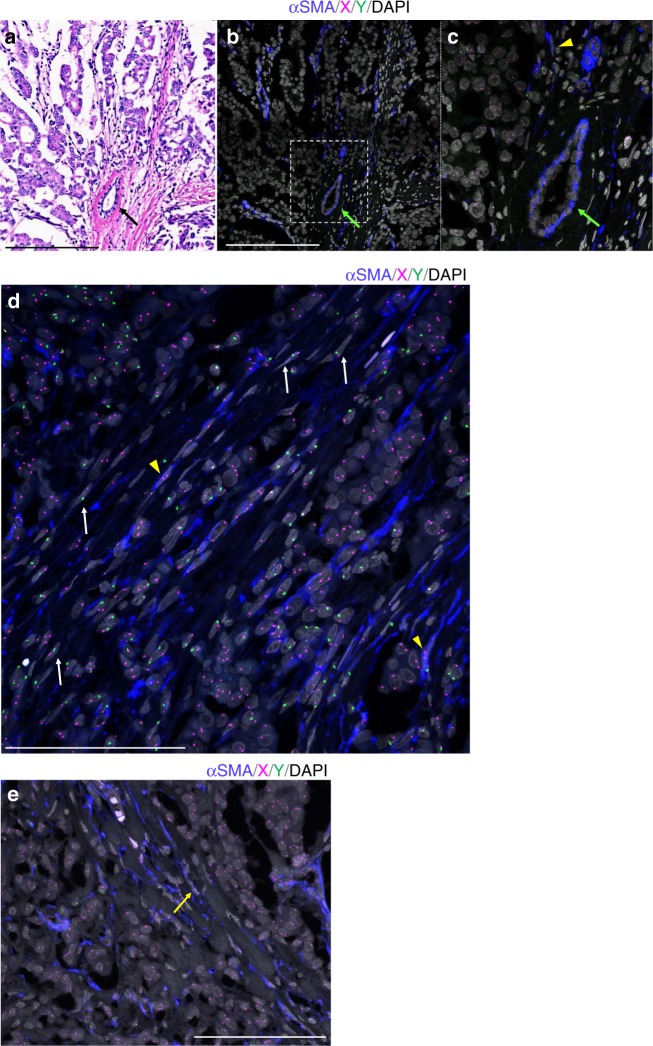
Case 1, breast carcinoma after sex-mismatched transplantation (male-to-female). **a** Hematoxylin and eosin (H&E) specimen of the invasive ductal carcinoma. Cancer cells form tubules or nests and infiltrate with involvement of a desmoplastic reaction. A normal mammary duct with an inner epithelial cell layer and an outer layer of myoepithelial cells is observed beside the tumor (black arrow). Scale bar, 200 µm. **b** α-Smooth muscle actin (αSMA) immunofluorescent in situ hybridization (ImmunoFISH) analysis of chromosomes X and Y performed on the same section as **a**. Immunostaining for αSMA highlights αSMA(+) spindle cells including cancer-associated fibroblasts (CAFs), pericytes of blood vessels, and myoepithelial cells of mammary ducts (green arrow). αSMA, blue; X, magenta; Y, green; DAPI, gray. Scale bar: 200 µm. **c** Magnified image of the inset to **b**. A recipient-derived αSMA(+) stromal cells with spindle-shaped nuclei (SCSSNs) is observed (yellow arrowhead). Compared with the H&E-stained image (**a**) obtained using a classical optical microscope, more nuclei are observed in this immunoFISH image. This is because this is a confocal microscope maximum intensity projection image of a *z*-stack; therefore, the nuclei deep in the section that could not be observed using a classical optical microscope could be seen. **d** Additional image of αSMA ImmunoFISH analysis. Representative donor-derived αSMA(−) SCSSNs are indicated by white arrows and recipient-derived αSMA(+) SCSSNs by yellow arrowhead. αSMA, blue; X, magenta; Y, green; DAPI, gray. Scale bar: 100 µm. **e** Additional image of αSMA ImmunoFISH analysis. Donor-derived αSMA(+) SCSSN is indicated by yellow arrow. αSMA, blue; X, magenta; Y, green; DAPI, gray. Scale bar: 100 µm

**Fig. 2:**
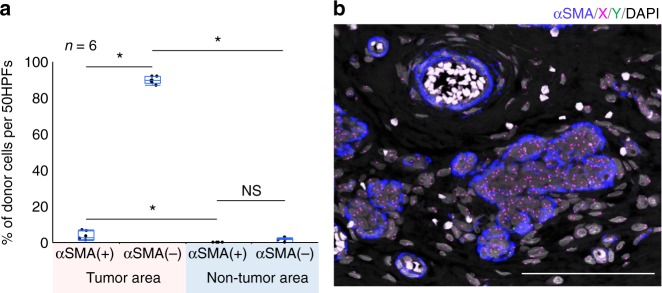
Case 1, breast carcinoma after sex-mismatched transplantation (male-to-female). **a** The proportion of donor SCSSNs in a region comprising 50 continuous HPFs (high-power fields, 1 HPF = 70,225 µm^2^) in a tumor and non-tumor area. Each analysis was carried out in six regions of two separate sections. Combined box-and-whisker and dot plots, which show medians (lines), 25th–75th percentiles (boxes), and the furthest points within 1.5× interquartile range from the box (whiskers). The interquartile range was calculated as the value in the third quartile minus that in the first quartile. The total numbers of cells were as follows: 608 αSMA(+) SCSSNs and 1533 αSMA(−) SCSSNs in the tumor area and 3 αSMA(+) SCSSNs and 359 αSMA(−) SCSSNs in the non-tumor area. *p < 0.05. NS not significant (Steel–Dwass test). **b** ImmunoFISH analysis of the non-tumor area. Most SCSSNs were recipient-derived. αSMA, blue; X, magenta; Y, green; DAPI, gray. Scale bar: 200 µm

**Fig. 3:**
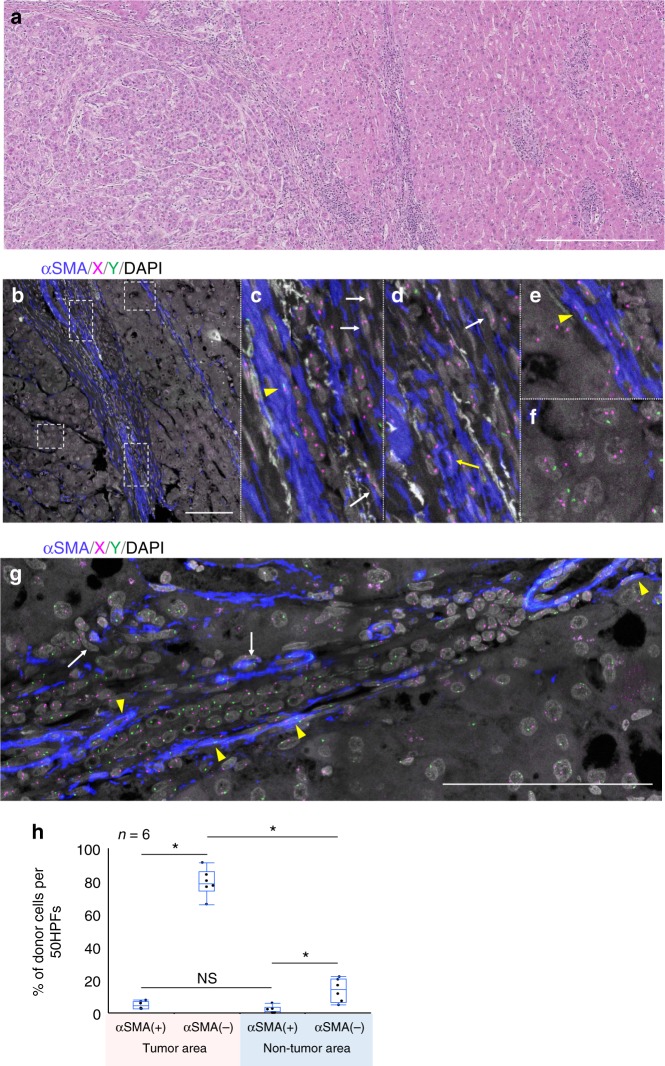
Case 2, hepatocellular carcinoma after sex-mismatched transplantation (female to male). **a** H&E specimen. On the left side of the picture, a hepatocellular carcinoma is observed; the non-tumor cirrhotic liver is on the right. Scale bar, 500 µm. **b**–**f** ImmunoFISH analysis of the area of the hepatocellular carcinoma. **c**–**f** Magnified images of the insets to **b**. Donor-derived αSMA(+) SCSSNs are indicated by yellow arrows and donor-derived αSMA(−) SCSSNs by white arrows. Recipient-derived αSMA(+) SCSSNs are indicated by yellow arrowheads. Tumor cells showing sex chromosome aneuploidy are evident in **f**. αSMA, blue; X, magenta; Y, green; DAPI, gray. Scale bar, 100 µm. **g** ImmunoFISH analysis of liver cirrhosis (non-tumor area). Donor-derived αSMA(−)SCSSNs are indicated using white arrows; recipient-derived αSMA(+) SCSSNs by yellow arrowheads. αSMA, blue; X, magenta; Y, green; DAPI, gray. Scale bar, 100 µm. **h** The proportions of donor SCSSNs in regions of the tumor and non-tumor areas. The analysis was carried out in six regions of three sections. The total numbers of cells analyzed were as follows: 315 αSMA(+) SCSSNs and 645 αSMA(−) SCSSNs in the tumor area and 213 αSMA(+) SCSSNs and 331 αSMA(−) SCSSNs in the non-tumor area. *p < 0.05. NS, not significant (Steel–Dwass test)

To assess the possibility of chimerism in stromal cells with spindle-shaped nuclei (SCSSNs), we performed immunoFISH, a combination of α-SMA immunostaining and dual-color FISH analysis of the X and Y chromosomes. In all cases, donor-derived SCSSNs were observed in the tumor stroma (Figs. 1–3 and Supplementary Figure 1). Compared with non-invasive tumors, donor-derived SCSSNs were observed more frequently in invasive tumors.

In case 1, a breast carcinoma from a 37-year-old female, infiltrating tumor cells exhibited a trabecular or glandular pattern, accompanied by desmoplastic reactions (Fig. 1a). ImmunoFISH analysis of this tumor was performed on the same section as shown in Fig. 1a, with maximum intensity projection images from the *z*-stack, using the ZEN software (Fig. 1b, c). The images presented below (immunoFISH or triple immunostaining images) are also maximum intensity projection images images of the *z*-stack. Compared with the original hematoxylin and eosin (H&E)-stained image (Fig. 1a), tumor structure and cell morphology were well preserved even after immunoFISH staining, to the point where it was possible to analyze tissue morphology using immunoFISH images alone. Tumor cells exhibited recipient-derived XX signals, compared with SCSSNs present in the desmoplastic stroma, which displayed donor-derived XY signals or recipient-derived XX signals (Fig. 1b–e). The mean donor chimerism rate of αSMA(+) SCSSNs was 3.7% (standard deviation (SD) 2.4%), compared with 89.7% (SD 2.0%) for αSMA(−) SCSSNs (Fig. 2a). In the non-tumor area, myoepithelial cells of the duct or terminal duct lobular units or the pericytes of the blood vessels were observed as αSMA(+) spindle cells, all of which were derived from the recipient (Fig. 2b). Evidence of other SCSSNs was scarce relative to that seen in the tumor area, with no donor-derived αSMA(+) SCSSNs found, and only a few donor-derived αSMA(−) SCSSNs present 1.3% (SD 1.1%) (Fig. 2a). Levels of both donor-derived αSMA(+) SCSSNs and αSMA(−) SCSSNs in the non-tumor area were substantially less than seen in the tumor area.

In case 2, a hepatocellular carcinoma of a 64-year-old male, the tumor cells revealed instances of both polyploidy and aneuploidy containing XY signals, indicating cells of recipient origin (Fig. 3f). Within the tumor stroma, few αSMA(+) SCSSNs and many αSMA(−) SCSSNs displayed donor XX signals (Fig. 3b–e, h), with donor chimerism seen in 4.6% (SD 2.4%) of αSMA(+) SCSSNs and 79% (SD 8.4%) of αSMA(−) SCSSNs. In the non-tumor area, liver cirrhosis with advanced fibrosis was seen and αSMA(+) SCSSNs were frequently observed, most of which were from the recipient (Fig. 3g, h). The donor chimerism rate of αSMA(+) SCSSNs was 1.3% (SD 2.3%), compared with 13.7% (SD 7.0%) for αSMA(−) SCSSNs.

In case 3, an oral squamous cell carcinoma arising at the buccal mucosa in a 66-year-old male, the tumor proliferation pattern was solid-like, and the tumor stroma was relatively poor (Supplementary Figure 1a–c). The donor chimerism rate of αSMA(+) SCSSNs was 1.2% (SD 1.8%), compared with 74.8% (SD 5.0%) for αSMA(−) SCSSNs. In the non-tumor area, the donor chimerism rate of αSMA(+) SCSSNs was 0%, compared with 6.1% (SD 5.2%) for αSMA(−) SCSSNs.

In cases 4–6, non-invasive gastrointestinal carcinomas and colon adenoma, only a handful of donor-derived SCSSNs were found in either the tumor or non-tumor areas (Supplementary Figure 1d–f). In each case, no donor-derived αSMA(+) SCSSNs were observed, and the donor chimerism rate of αSMA(−) SCSSNs was <5%.

### Contribution of cell fusion to donor-derived SCSSNs

To determine whether cell fusion occurred, the number of sex chromosome signals in the nuclei of the SCSSNs was counted. Cell fusion-derived cells typically have four chromosomes. However, in 5 μm-thick histological sections, loss of part of the nucleus occurs at a certain frequency (i.e., cutting artifact); hence, some of the cells with four sex chromosomes have 0–3 sex chromosome signals. Even if derived from cell fusion, cells with only 0–2 sex chromosome signals may appear not to be derived from cell fusion (i.e., a false negative). Cells with three or four sex chromosome signals were thus interpreted as being derived from cell fusion. To estimate the probability of correct judgement, X/Y FISH analysis of spindle cell tumors with four sex chromosome signals per cell is required. However, because we did not have any such specimens, we instead used oral mucosa scar tissue, which is composed of normal spindle-shaped stromal cells with two sex chromosomes. We used the probability of spindle-shaped cells with two sex chromosome signals in scar tissue as a rough estimate of the probability of correct judgement. X/Y FISH analysis of scar tissue revealed that 292 of the 500 spindle-shaped cells had two signals, and the probability of correct judgment was 58%.

In the above immunoFISH analysis of case 1, the sex chromosome signals of donor-derived SCSSNs were also enumerated. All donor-derived αSMA(+) SCSSNs (24 total) in 300 high-power fields (HPF, 1 HPF = 70,225 µm^2^) and 500 donor-derived αSMA(−) SCSSNs were analyzed. Among the donor-derived αSMA(+) SCSSNs and donor-derived αSMA(−) SCSSNs, 0 and 3 had ≥3 signals, respectively (both <1%). Even if all of these cells were derived from cell fusion, the actual proportion of cell fusion-derived cells was estimated to be ≤1%. These results suggest that cell fusion did not contribute significantly to the generation of the SCSSNs.

### Contribution of bone marrow stromal cells to SCSSNs

Next, we sought to determine whether bone marrow-derived SCSSNs originated from hematopoietic cells or bone marrow stromal cells. As SCSSNs derived from hematopoietic cells would be considered macrophages or dendritic cells based on their morphology, either HLA-DR or CD68 could be used as a common marker^35^. On the other hand, SCSSNs derived from bone marrow mesenchymal cells would be consistent with that of bone marrow stromal cells, such as mesenchymal stem cells or pericytes, making platelet-derived growth factor receptor beta (PDGFRβ) the preferred common marker^36^. HLA-DR and PDGFRβ were therefore chosen as markers for downstream analyses.

**Fig. 4:**
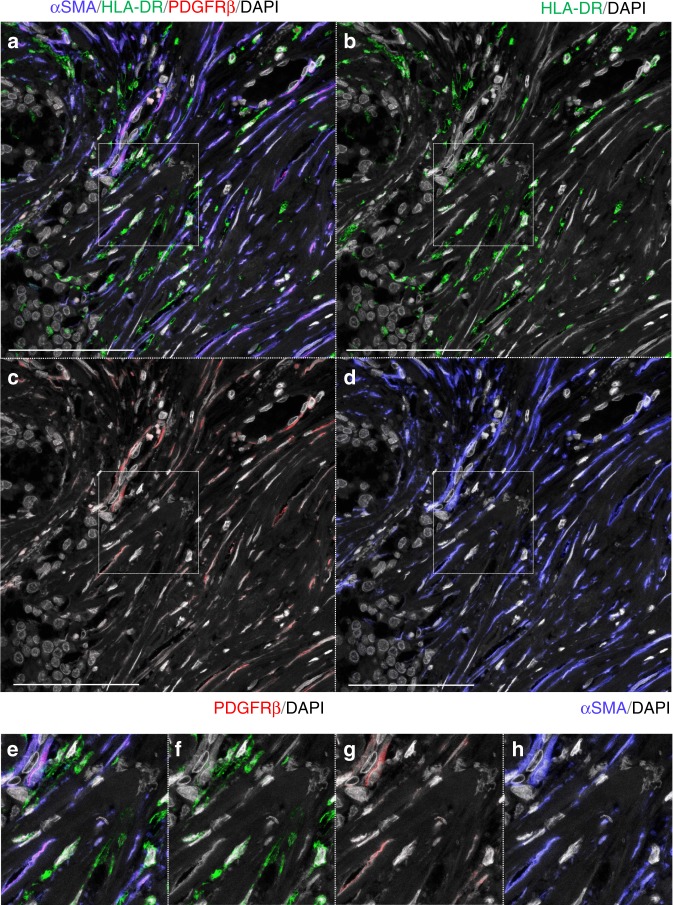
Triple immunostaining using markers of bone marrow stromal cell in case 1. αSMA, blue; PDGFRβ, red; HLA-DR, green; DAPI, gray. **a**–**d** Triple immunostaining with αSMA, HLA-DR, and PDGFRβ. αSMA(+)SCSSNs were almost identical to PDGFRβ(+)SCSSN (mean 99.3% (SD 0.6%) of αSMA(+)SCSSNs). and αSMA(+)HLA-DR(+) SCSSNs were sparse (mean 0.9% (SD 0.7%) of αSMA(+)SCSSNs). The analysis was carried out in six regions that consist of 5 HPFs (total 722 αSMA(+)SCSSNs). Scale bar: 100 μm. **e**–**h** Magnified images of the insets in **a**–**d**

**Fig. 5:**
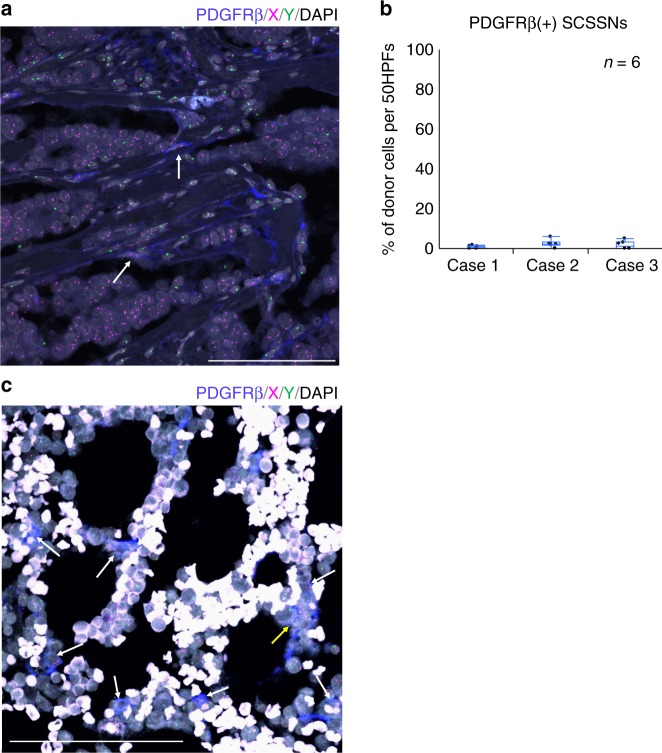
ImmunoFISH for bone marrow stromal cell markers in case 1. PDGFRβ, blue; X, magenta; Y, green; DAPI, gray. **a** PDGFRβ-immunoFISH analysis in the tumor specimen. Recipient-derived PDGFRβ(+) SCSSNs and donor-derived PDGFRβ(−) SCSSNs are observed. Recipient-derived PDGFRβ(+) SCSSNs are indicated by white arrows. Scale bar: 100 μm. **b** Proportions of donor cells in a region comprising 50 continuous HPFs. Each analysis was carried out in six regions of the tumor area. The means (SD, total number of PDGFRβ(+) SCSSNs, number of sections) were as follows: 0.7% (0.8%, 578, 2) in case 1, 2.4% (1.9%, 258, 3) in case 2, and 1.8% (2.1%, 182, 2) in case 3. (PDGFRβ-immunoFISH images for cases 2 and 3 are shown in Supplementary Figs. 2 and 3). **c** PDGFRβ-immunoFISH analysis in the bone marrow specimen. Most PDGFRβ(+) cells are recipient-derived (white arrow). Cells marked weakly positive for PDGFRβ (weak+) are suspected to be donor-derived (yellow arrow). Scale bar: 100 μm

Paraffin sections of three invasive carcinomas (cases 1–3) were triple immunostained with antibodies targeting αSMA, HLA-DR, and PDGFRβ. Greater than 96% of αSMA(+) cells stained positive for PDGFRβ and negative for HLA-DR, compared with virtually no PDGFRβ(+) αSMA(−) SCSSNs (Fig. 4, Supplementary Figures 2a–d and 3a–d). Furthermore, PDGFRβ-immunoFISH analysis revealed only a small number of donor-derived PDGFRβ(+) cells (0.7–2.4%) in these samples (Fig. 5a, b. Supplementary Figures 2e and 3e).

In conventional human allogeneic stem cell transplantation, only a small proportion of mesenchymal stem cells are able to sustain long-term engraftment in the recipient’s bone marrow^37^. If donor-derived αSMA(+) PDGFRβ(+) SCSSNs were to have originated from bone marrow stromal cells, donor-derived stromal cells should have been engrafted in the bone marrow. To address this question, we performed a chimerism analysis using PDGFRβ as a marker on a bone marrow specimen. Case 1 was selected for this assay because of the availability of bone-marrow histological specimens 3 years after bone marrow transplantation (Fig. 5c). PDGFRβ was chosen as a marker due to its strong expression in bone marrow stromal cells, pericytes, and osteoblasts, while being negative for hematopoietic cells other than eosinophils. Further differentiation between eosinophils and other stromal cells was determined based on auto-fluorescence. Staining on two serial sections revealed that 471 of the 500 (94%) nucleated cells were donor-derived, while 86% of recipient-derived cells were PDGFRβ(+) cells. Donor-derived PDGFRβ(+) cells were also detected, accounting for 7% of 200 PDGFRβ(+) cells; however, the vast majority of this population (6%) was presumed to be eosinophils, while the remaining 1% were only weakly positive, and typically of uncertain origin. Because there were much fewer αSMA(+) cells than PDGFRβ(+) cells in the bone marrow specimens, a detailed analysis of αSMA(+) cell chimerism was difficult. This result suggests that donor-derived stromal cells, including mesenchymal stem cells, may not have established long-term engraftment.

### Many αSMA(−) SCSSNs were macrophages and dendritic cells

**Fig. 6:**
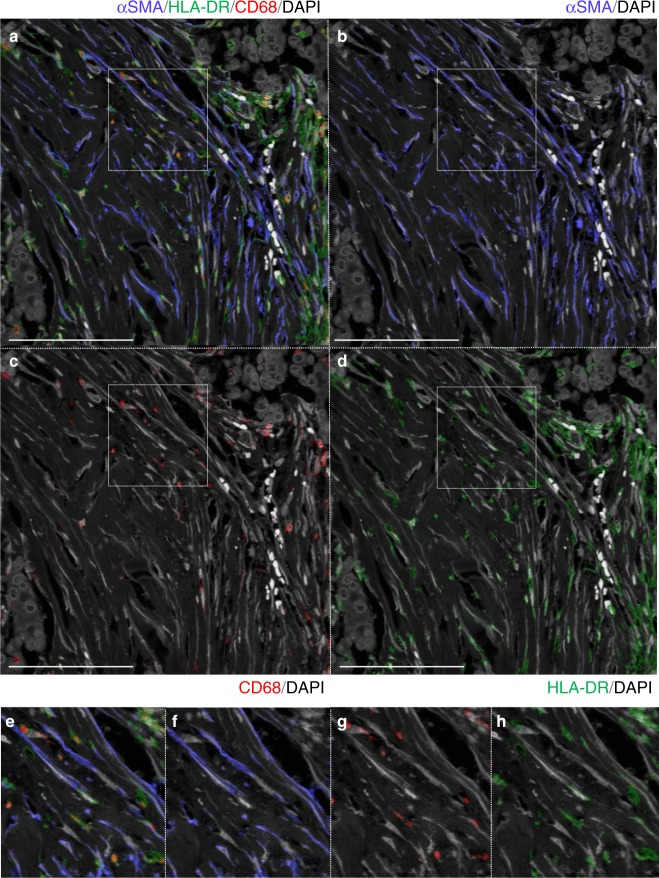
Triple immunostaining for macrophage and dendritic cell markers in case 1. αSMA, blue; CD68, red; HLA-DR, green; DAPI, gray. **a**–**d** Triple immunostaining for αSMA, HLA-DR, and CD68. 88% of the 136 αSMA(−) SCSSNs were HLA-DR(+) and 84% of the 119 HLA-DR(+) SCSSNs expressed CD68 at various intensities (in 5 HPFs). Scale bar: 100 μm. **e**–**h** Magnified images of the insets in **a**–**d**

**Fig. 7:**
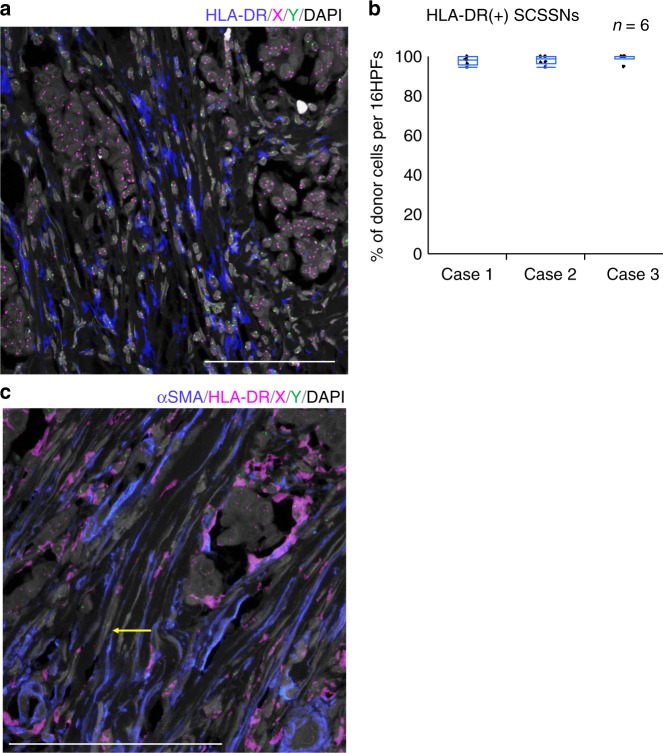
ImmunoFISH for macrophage and dendritic cell markers in case 1. **a** HLA-DR-immunoFISH. Most HLA-DR(+)SCSSNs originated from the donor. HLA-DR, blue; X, magenta; Y, green; DAPI, gray. Scale bar: 100 μm. **b** The proportions of donor cells in a region comprising 16 continuous HPFs. Each analysis was carried out in six regions of the tumor area in cases 1–3. The mean (SD, total number of HLA-DR(+) SCSSNs, number of sections) proportions of donor cells were as follows: 97.8% (2.2,% 325, 2) in case 1, 98.1% (2.3%, 156, 2) in case 2, and 99.1% (2.1%, 115, 2) in case 3. HLA-DR-immunoFISH images from cases 2 and 3 are shown in Supplementary Figures 4 and 5. **c** ImmunoFISH for αSMA and HLA-DR. Donor-derived αSMA(+) HLA-DR(−) SCSSNs are observed; Donor-derived αSMA(−) HLA-DR(−) SCSSN is indicated by yellow arrow. αSMA, blue; HLA-DR, magenta; X, magenta; Y, green; DAPI, gray. Scale bar, 50 μm

Next, we examined the origin of αSMA(−) SCSSNs. From the above-mentioned results, the contribution of bone marrow stromal cells to αSMA(−) SCSSNs was considered to be very low. If αSMA(−) SCSSNs were to have originated from bone marrow hematopoietic cells, macrophages or dendritic cells were considered likely candidates based on cell morphologically. We therefore investigated the percentage of macrophages or dendritic cells in αSMA(−) SCSSNs by triple immunostaining for αSMA, CD68, and HLA-DR using CD68 and HLA-DR as common markers for macrophages and dendritic cells. Of the αSMA(−) SCSSNs, 73–88% tested positive for HLA-DR, with the majority of HLA-DR(+) cells also expressing a moderate-to-strong level of CD68, indicating that αSMA(−) HLA-DR(+) CD68(+) SCSSNs were derived from macrophages or dendritic cells (Fig. 6, Supplementary Figures 4a–d and 5a–d). αSMA(+) HLA-DR(+) SCSSNs were rarely observed, and it was not clear whether the cytoplasm of the two SCSSNs overlapped or whether αSMA and HLA-DR were truly co-expressed. Furthermore, chimerism analysis using immunoFISH revealed that virtually all HLA-DR(+) SCSSNs were donor-derived (Fig. 7a, b, Supplementary Figures 4e and 5e). In addition, a few donor-derived αSMA(−) HLA-DR(−) SCSSNs were observed by immunoFISH via double staining of αSMA and HLA-DR (Fig. 7c). From these results, it can be concluded that the majority of αSMA(−) SCSSNs were derived from hematopoietic cells, primarily macrophages or dendritic cells, with the origin of less common HLA-DR(+) CD68(−) SCSSNs with unknown differentiation likely mixed.

### CD163(+) macrophages were derived from the bone marrow

**Fig. 8:**
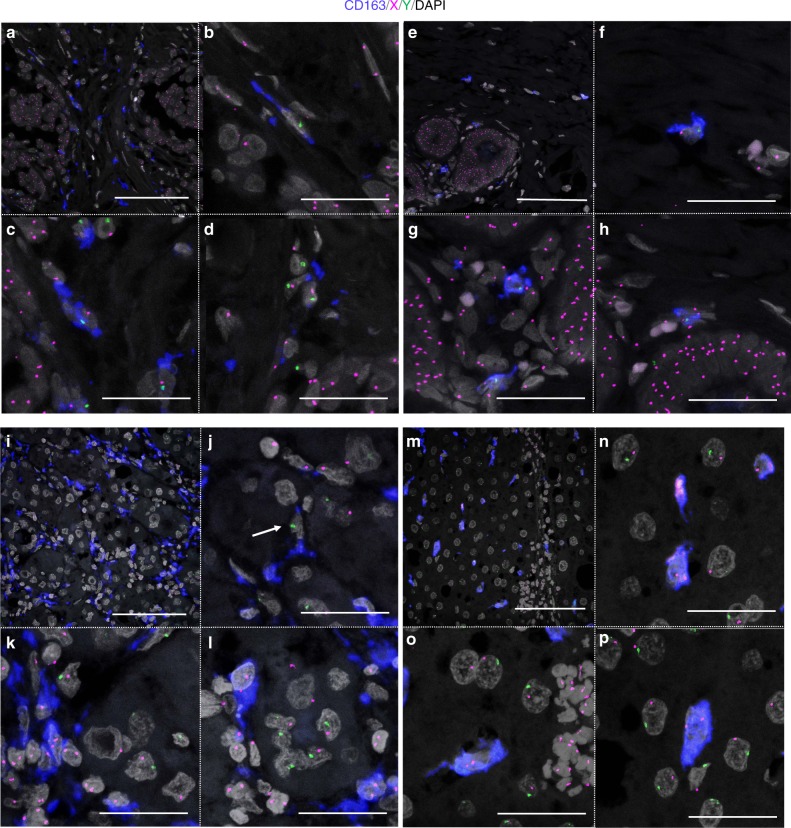
CD163-immunoFISH analysis in tumor and non-tumor tissues in cases 1 and 2. CD163, blue; X, magenta; Y, green; DAPI, gray. **a**–**h** Breast carcinoma in a 37-year-old female. **a**–**d** Tumor area. **b**–**d** Magnified images of **a**. **e**–**h** Non-tumor area. **f**–**h** Magnified images of **e**. Scale bars, **a**, **e** 100 μm and **b**–**d**, **f**–**h** 50 μm. **i**–**p** Hepatocellular carcinoma in a 64-year-old male. **i**–**l** Tumor area. **j**–**l** Magnified images of **i**. **j** The cell indicated by white arrow looks like a recipient-derived macrophage. However, when examined in the *z*-stack, recipient-derived CD163(−) cell and CD163(+) macrophages without the sex chromosome due to cutting artifact were overlapped in the *z* direction. **m**–**p** Non-tumor area. **n**–**p** Magnified images of **m**. Scale bars: **i**, **m** 100 μm and **j**–**l**, **n**–**p** 50 μm

**Fig. 9:**
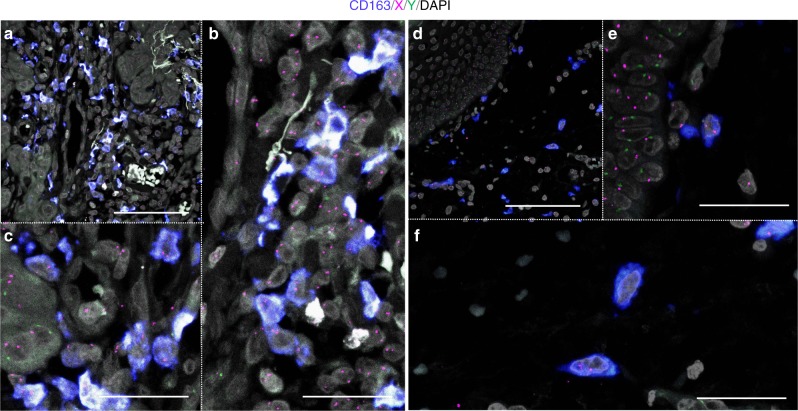
CD163-immunoFISH images of tumor and non-tumor tissues in case 3. Oral squamous cell carcinoma in a 66-year-old male. CD163, blue; X, magenta; Y, green; DAPI, gray. **a**–**c** Tumor area. **b**, **c** Magnified images of **a**. Scale bars: **a** 100 μm and **b**, **c** 50 μm. **d**–**f** Non-tumor area. **e**, **f** Magnified images of **d**. Scale bars, **d** 100 μm and **e**, **f** 50 μm

The majority of macrophages originate from bone marrow-derived monocytes or yolk-sac progenitors. In mice, TAMs are reported to be derived from the bone marrow^26–28^. To investigate the extent to which bone marrow-derived cells contribute to macrophage development in human tissues, we performed CD163-immunoFISH analysis on tumor and non-tumor tissue specimens from three cases of invasive carcinoma (cases 1–3). CD163 was chosen based on its relatively high specificity for macrophages^35^. For cases 1 and 3, non-tumor tissue specimens were chosen from areas without inflammation; such an analysis could not be performed for case 2 as the non-tumor area was liver cirrhosis. In all three cases, almost all CD163(+) macrophages were found to be donor-derived not only in the tumor but also in the non-tumor tissue (Figs. 8 and 9, Supplementary Table 1). The main distinction between these two locations was evident based on cell morphology, with CD163(+) macrophages in the non-tumor area exhibiting oval or ellipsoidal but not spindle-shaped nuclei, while the nuclei of tumor-resident CD163(+) macrophages exhibited various shapes, such as oval, ellipsoid, and spindle.

## Discussion

To verify the presence of bone marrow-derived CAFs in human cancer, we performed chimerism analysis of CAFs using histopathological specimens of secondary tumors that developed after sex-mismatched allogeneic stem cell transplantation. As CAFs are heterogeneous and lack a specific marker, we analyzed SCSSNs in tumor tissue as possible candidates for CAFs. Moreover, we performed a chimerism analysis of TAMs and macrophages in non-tumor tissues by immunoFISH, allowing simultaneous detection of immunophenotypic markers and sex chromosomes on a single paraffin section. These efforts were aided by the use of a confocal laser microscope, making it possible to stereoscopically observe tissue construction at the cellular level, enabling more precise assessment of tissue chimerism. Beyond this, we developed a method to compare H&E images with immunoFISH images obtained from a single paraffin section, including assays in which immunoFISH was used to combine multiple markers. Using these approaches, we obtained the following results in three cases of invasive carcinoma. (1) Many bone marrow-derived SCSSNs were observed in the tumor stroma. (2) The possibility that cell fusion occurred in SCSSNs was extremely low. (3) Most αSMA(+) SCSSNs were derived from recipients. (4) Almost all bone marrow stromal cells were derived from recipients. (5) Many αSMA(−) SCSSNs, which are typically similar to fibroblasts, were actually bone marrow-derived HLA-DR(+) cells, the majority of which are macrophages or dendritic cells. (6) All CD163(+) macrophages were derived from bone marrow in both tumor as well as non-tumor tissues.

To avoid false-positive signals due to adjacent or overlapping cells in our chimerism analysis, only cells for which nuclei were surrounded by positive signal were judged as positive. In infiltrating carcinomas, many donor-derived SCSSNs were observed in the cancer stroma. Many αSMA(−) SCSSNs were donor in origin, although the vast majority of αSMA(+) SCSSN were recipient in origin, with substantially lower rates of donor chimerism than previously reported in mouse experiments^4–6,10^.

ImmunoFISH was also used to determine whether cell fusion had occurred using XY probes. Under normal circumstances, all SCSSNs would be expected to have up to two sex chromosome signals (XX or XY); if cell fusion had occurred, ≥3 sex chromosome signals (i.e., XXXY) may be observable. However, in our system, due to cutting artifacts, cell fusion-derived cells may exhibit two or fewer signals (i.e., a false negative). We calculated the probability of correct judgment by X/Y FISH analysis of non-tumor scar tissue and applied it to tumor tissue. In tumor tissue, <1% of donor-derived αSMA(−) SCSSNs exhibited ≥3 signals and thus may comprise both cell fusion-derived cells and cells undergoing fission. Even if all of these cells were derived from cell fusion, the actual proportion of cell fusion-derived cells was estimated to be ≤1%. Therefore, cell fusion did not make a considerable contribution to the generation of donor-derived αSMA(−) SCSSNs. For donor-derived αSMA(+) SCSSNs, because the total number of analyzable cells was small, the possibility of cell fusion cannot be ruled out. However, cells with ≥3 signals were not identified, suggesting that cell fusion did not contribute to the generation of donor-derived αSMA(+) SCSSNs (<1%).

Having established the respective origins of αSMA(+) and αSMA(−) SCSSNs, we next sought to identify the bone marrow populations from which these cells were derived. Cells were identified using HLA-DR and CD68 as common markers of macrophages and dendritic cells^35^, and PDGFRβ as a marker of various mesenchymal cell populations in bone marrow^36^. Nearly all αSMA(+) SCSSNs were found to be HLA-DR(−) CD68(−) PDGFRβ(+). Based on these results, if bone marrow-derived cells were present in αSMA(+) SCSSNs, they would likely have arisen from bone marrow stromal cells. In that case, the bone marrow stromal cells should have existed in a mixed chimeric state, with recipient cells partially substituted by donor-derived cells. However, for most human bone marrow transplantations, stromal cells, including mesenchymal stem cells, do not establish long-term engraftment^37^. On the other hand, several mouse experiments have shown that mesenchymal stem cells can be replaced by donor-derived cells after bone marrow transplantation^10,38^, suggesting the possibility of long-term stromal cell engraftment in humans.

Next, we performed chimerism analysis of PDGFRβ(+) cells in bone marrow tissues collected 3 years after bone marrow transplantation. As a result, the proportion of cells considered to be donor-derived PDGFRβ(+) stromal cells was only ~1%, with most staining weak and potentially non-specific. The number of mesenchymal stem cells in bone marrow is typically very small, accounting for only 0.001–0.01% of the total nucleated cells^39^. The precise chimerism rate of mesenchymal stem cells in this study could not be determined, as the majority of PDGFRβ expression in bone marrow is associated with more differentiated stromal cells, of which mesenchymal stem cells are only a minor constituent. However, based on the chimerism of PDGFRβ(+) stromal cells that would have differentiated from mesenchymal stem cells, the possibility of donor-derived mesenchymal stem cells' engraftment is considered very low. As mentioned above, we observed only a small number of donor-derived αSMA(+) SCSSNs in cancer stroma in contrast to previous reports. One reason for this difference may be the poor engraftment of donor-derived bone marrow stromal cells. It is not known whether recipient-derived αSMA(+) SCSSNs also contained recipient-derived bone marrow stromal cells or whether the contribution of bone marrow stromal cells to CAFs was simply limited.

As for the fate of donor-derived αSMA(+) SCSSNs, we hypothesize that donor-derived αSMA(−) SCSSN entered the cytoplasm of αSMA(+)SCSSNs (false positive), (2) donor-derived bone marrow stromal cells engrafted in a region other than the ilium were recruited to the tumor site, and (3) the possibility of cell fusion could not be completely ruled out.

For αSMA(−) SCSSNs, many cells were observed in the cancer stroma, including numerous cells with spindle-shaped nuclei that most closely resembled fibroblasts. Among αSMA(−) SCSSNs, 74.8–89.7% were derived from bone marrow, and the majority of them were revealed to be HLA-DR(+) CD68(+) macrophages or dendritic cells by multiple staining and chimerism analysis. A recent study in mice showed that monocyte/macrophage-derived αSMA(+) cells formed a hematopoietic stem cell niche in the bone marrow^40^. Beyond these cell populations, we considered the possibility that TAMs may differentiate into αSMA(+) SCSSNs. However, in our study, almost all αSMA(+) SCSSNs were PDGFRβ(+) HLA-DR(−), which is the opposite expression pattern for macrophages and dendritic cells, indicating that TAMs are highly unlikely to differentiate into αSMA(+) SCSSNs.

To investigate the extent to which bone marrow-derived macrophages were present in human tumor or non-tumor tissues, we performed a chimerism analysis of CD163(+) cells in three cases of invasive carcinoma that developed 3.6–19.8 years after bone marrow transplantation. In all three cases, the vast majority of CD163(+) cells in tumor tissues were derived from bone marrow. Not all TAMs are CD163(+)^41^. However, because almost all HLA-DR(+) macrophages or dendritic cells were derived from the bone marrow, most TAMs were also likely derived from the bone marrow. This is consistent with the results of mouse studies, in which TAMs were found to originate from bone marrow-derived inflammatory monocytes^26–28^.

Furthermore, in non-tumor tissues (mammary gland, liver, and oral mucosa), almost all CD163(+) cells were derived from the bone marrow. Because macrophages and monocytes are positive for CD163, some of the CD163(+) cells should have been circulating monocytes. However, because all large and mature CD163(+) cells (Figs. 8 and 9) were also derived from the bone marrow, tissue macrophages had been completely replaced by bone marrow-derived cells in our cases. This is consistent with previous studies in bone marrow transplantation patients^29,30^. The differences compared with mouse and human organ transplant cases may be explained by the following reasons. (1) Humans have a longer lifespan than those of other animals and are not always in steady state, and thus macrophages derived from the yolk sac may be gradually replaced by bone marrow-derived macrophages. (2) The effects of whole-body irradiation and chemotherapy at the time of bone marrow transplantation may play a role. (3) In bone marrow transplantation, unlike organ transplantation, immunosuppressive drugs are generally tapered from 6 to 12 months after transplantation unless graft-versus-host disease develops. Donor-derived immune cells may have eliminated the progenitors of recipient-derived tissue macrophages.

## Methods

### Patients

Formalin-fixed paraffin-embedded tumor samples were selected from the archives of the Department of Pathology, Japanese Red Cross Nagoya First Hospital. Detailed information regarding each patient is listed in Table 1. The study was approved by the institutional review boards of Osaka University Hospital (No. 16127) and Japanese Red Cross Nagoya First Hospital (No. 2016–051).

### ImmunoFISH

ImmunoFISH was performed using the protocol of Bzorek et al.^42^. Briefly, 5 μm-thick formalin-fixed paraffin-embedded sections were blocked and incubated with a primary anti-human antibody against αSMA (1A4, 1:100, DAKO, Tokyo, Japan), CD68 (PG-M1, 1:100, DAKO), CD163 (10D6, 1:100, Novocastra, Newcastle, UK), PDGFRβ (28E1, 1:50, Cell Signaling Technology, Danvers, MA, USA), or HLA-DR (TAL1B5, 1:100, Abcam, Cambridge, UK) followed by incubation with the goat anti-mouse or anti-rabbit horseradish peroxidase conjugate included in the Tyramide Signal Amplification Kit (Thermo Fisher Scientific, Waltham, MA, USA). Signal amplification was performed using tyramide conjugated to Alexa Fluor 647 or 594. Multiple immunostainings were performed using the Tyramide Signal Amplification Kit with tyramide conjugated to Alexa Fluor 488, 594, or 647, according to the manufacturer’s protocol. Next, FISH was performed using CEP X SpectrumOrange/Y SpectrumGreen DNA probes (Vysis, Downers Grove, IL, USA). The slides were denatured and hybridized to the probes in hybridizer solution (DAKO). Next, the slides were stringently washed and counterstained with 4′,6-diamidino-2-phenylindole (DAPI) (Nacalai Tesque, Kyoto, Japan). Using the Zeiss LSM 710 or LSM 880 confocal microscope and ZEN microscope software (Carl Zeiss, Thornwood, NY, USA), confocal *z*-stack serial optical sections at 0.6 µm steps were captured across the entire thickness (5 µm) of the histological specimen under an ×40/1.3 NA oil-immersion objective in four fluorescence channels.

To obtain H&E- and immunoFISH-stained images from a single paraffin section, we first captured images of H&E-stained sections using a virtual microscope (×40 objective, NanoZoomer, Hamamatsu Photonics KK, Hamamatsu, Japan). We proceeded with the immunostaining protocol of Bzorek et al., as described above, except using H&E-stained slides instead of unstained slides. Using this method, the H&E stain is lost at the time of completion of antigen retrieval, enabling dual imaging of a single section.

### Chimerism analysis

To determine the proportion of donor-derived CAFs in each sample, a chimerism analysis was performed by stereoscopic analysis of individual cells via *z*-stack imaging. Using this approach, we counted the number of SCSSNs containing two FISH signals (XX or XY) in the tumor or within 0.5 HPFs of the tumor margin. To minimize the occurrence of false-positive results due to adjacent or overlapping cells, only cells with nuclei surrounded by a positive signal were judged as positive. The following stromal cells were not defined as CAFs: (1) cells with only one FISH signal (X or Y) due to cutting artifacts, and (2) cells involved in the formation of the lumen of vessels (glands, blood vessels, and lymphatic vessels). A certain number of consecutive HPFs were considered one region and the mean and SD rate of donor chimerism for multiple regions was calculated. For example, in the analysis of the chimerism rate for αSMA(+) SCSSNs, few cells per HPF had an αSMA signal around the nucleus with two sex chromosome signals. Therefore, we regarded 50 consecutive HPFs as one region and calculated the proportion of donor-derived cells in that region. The mean and SD rate of donor chimerism of six regions (*n* = 6) was calculated.

### Immunostaining analysis

Immunostaining was judged as positive when the immunostaining signal was in contact with the nucleus. In triple immunostaining analyses, co-expression was defined as two antibody signals in contact with one nucleus.

### Statistical analysis

The chimerism data are presented as combined box-and-whisker and dot plots, which show medians (lines), 25th–75th percentiles (boxes), and the furthest points within 1.5× interquartile range from the box (whiskers). The interquartile range was calculated as the value in the third quartile minus that in the first quartile. Steel–Dwass nonparametric multiple comparison test was used for comparison among groups. All statistical analyses were performed using the JMP software (SAS Institute Inc., Cary, NC, USA).

## Electronic supplementary material

Supplementary Information

## Data Availability

The data supporting the findings of this study are available within the manuscript or from the corresponding author upon reasonable request.
