# Supplementary material for: Origin of cancer-associated fibroblasts and tumor-associated macrophages in humans after sex-mismatched bone marrow transplantation
Source: Commun Biol. 2018 Sep 3;1:131. doi: 10.1038/s42003-018-0137-0 (PMC6123637; doi:10.1038/s42003-018-0137-0)
Supplement: Supplementary file 1 — Supplementary Information [file 42003_2018_137_MOESM1_ESM.pdf]

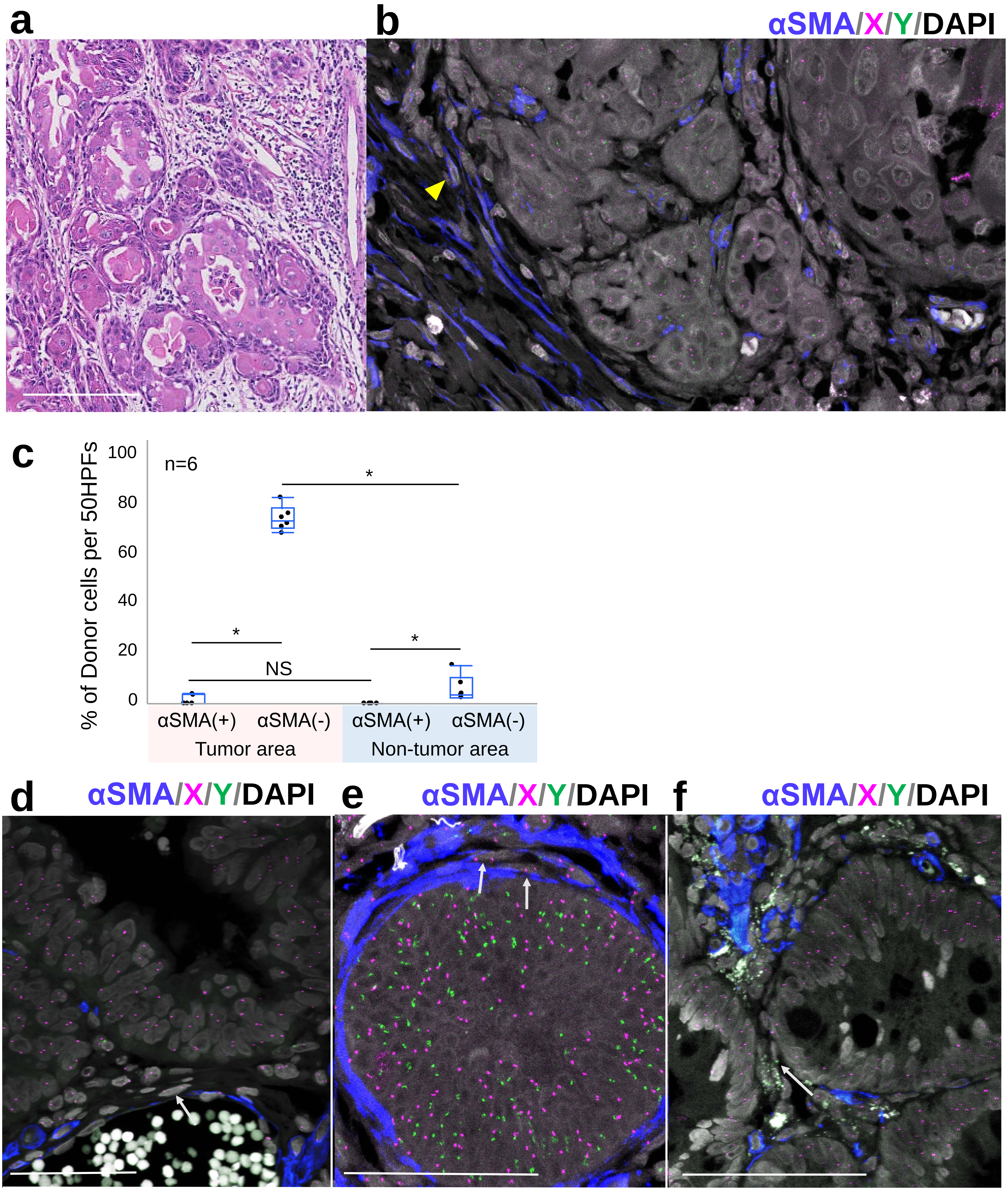

Supplementary Fig. 1. αSMA-ImmunoFISH images of chromosomes X and Y in cases 3–6. αSMA, blue; X, magenta; Y, green; DAPI, gray.
a, H&E specimen of case 3. Scale bar, 200 µm.
b, ImmunoFISH image of case 3. Recipient-derived αSMA(+) SCSSN is indicated by yellow arrowhead. Scale bar, 50 µm.
c, Proportions of donor SCSSNs in six regions of two sections of the tumor and non-tumor areas, as in Fig. 2. The total numbers of cells were as follows: 167 αSMA(+) SCSSNs and 454 αSMA(-) SCSSNs in the tumor area and 1 αSMA(+) SCSSN and 327 αSMA(-) SCSSNs in the non-tumor area. *p<0.05. NS, not significant (Steel–Dwass).
d, Case 4. Gastric adenocarcinoma in a 67-year-old male. Donor-derived αSMA(-) SCSSN is indicated by white arrow. Scale bar, 50 µm.
e, Case 5. Colon adenocarcinoma in a 68-year-old male. Donor-derived αSMA(-) SCSSNs are indicated by white arrows. Scale bar, 50 µm.
f, Case 6. Colon adenoma with high grade dysplasia in a 58-year-old female. Donor-derived αSMA(-) SCSSN is indicated by white arrow. Scale bar, 50 µm.

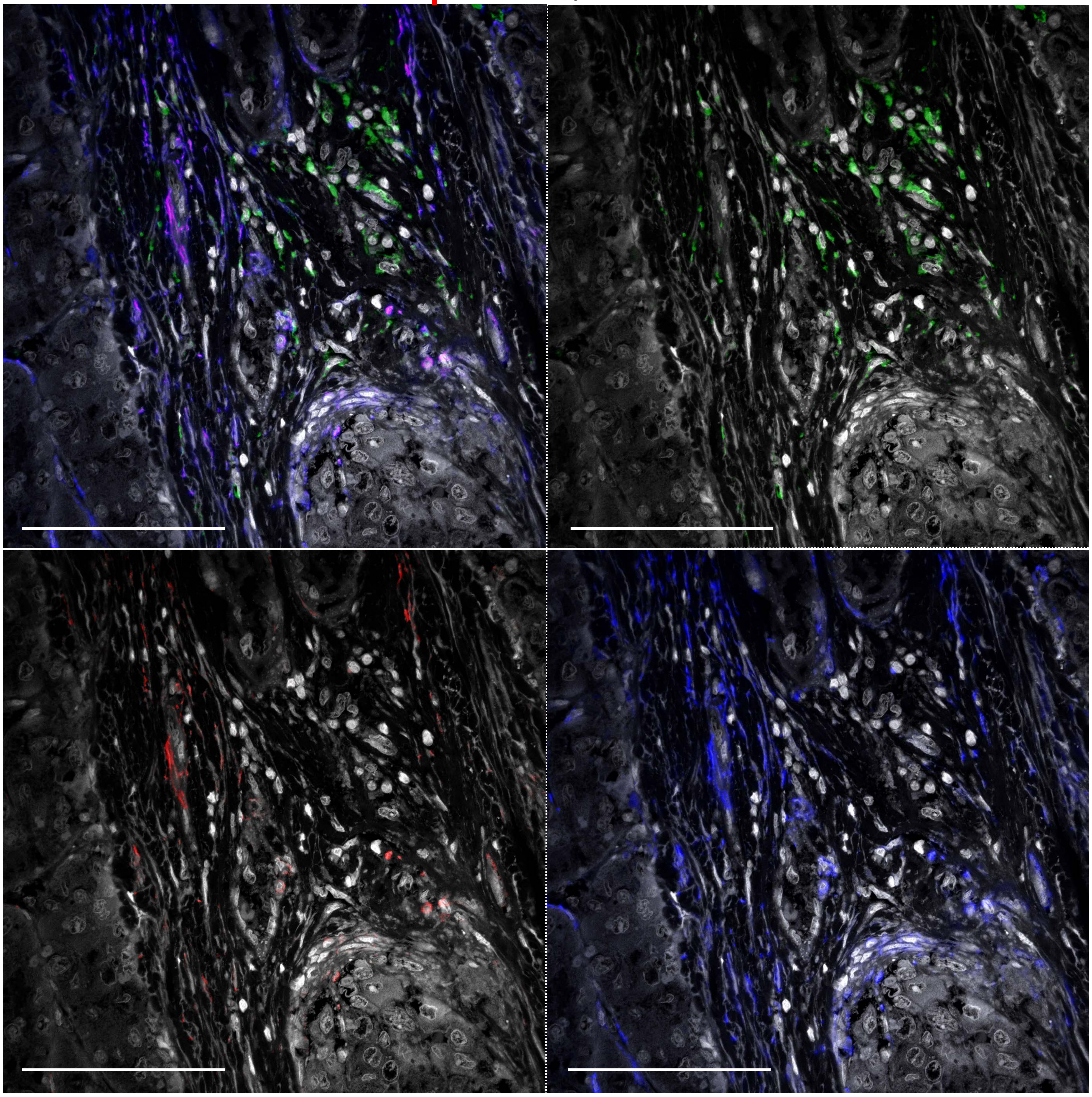

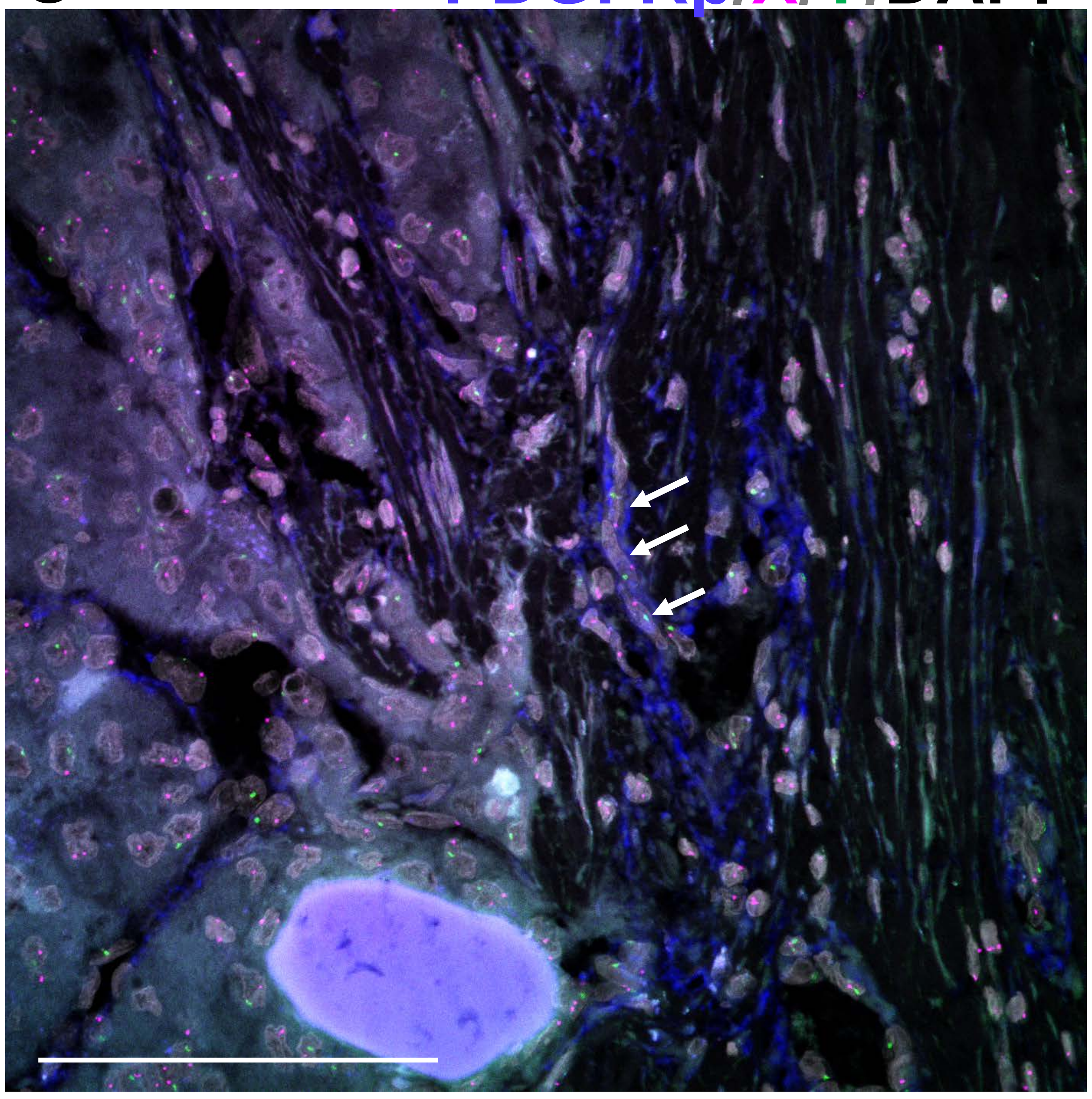

Supplementary Fig. 2 Triple immunostaining and immunoFISH for bone marrow stromal cell markers in case 2.
a–d, Triple immunostaining for αSMA, HLA-DR, and PDGFRβ. 96% of 106 αSMA(+) SCSSNs in 5 HPFs co-expressed PDGFRβ. PDGFRβ(+) αSMA(-) SCSSNs and αSMA(+) HLA-DR(+) SCSSNs were sparse (< 1% of the PDGFRβ(+)SCSSNs, αSMA(+) in 5 HPFs, respectively; > 100 cells were analyzed). PDGFRβ, blue; X, red; Y, green; DAPI, gray. Scale bar, 100 μm.
e, PDGFRβ-immunoFISH analysis in the tumor specimen. Recipient-derived PDGFRβ(+) cells are indicated by white arrows. The proportions of donor cells are shown in Fig. 5b. PDGFRβ, blue; X, magenta; Y, green; DAPI, gray. Scale bar, 100 μm.

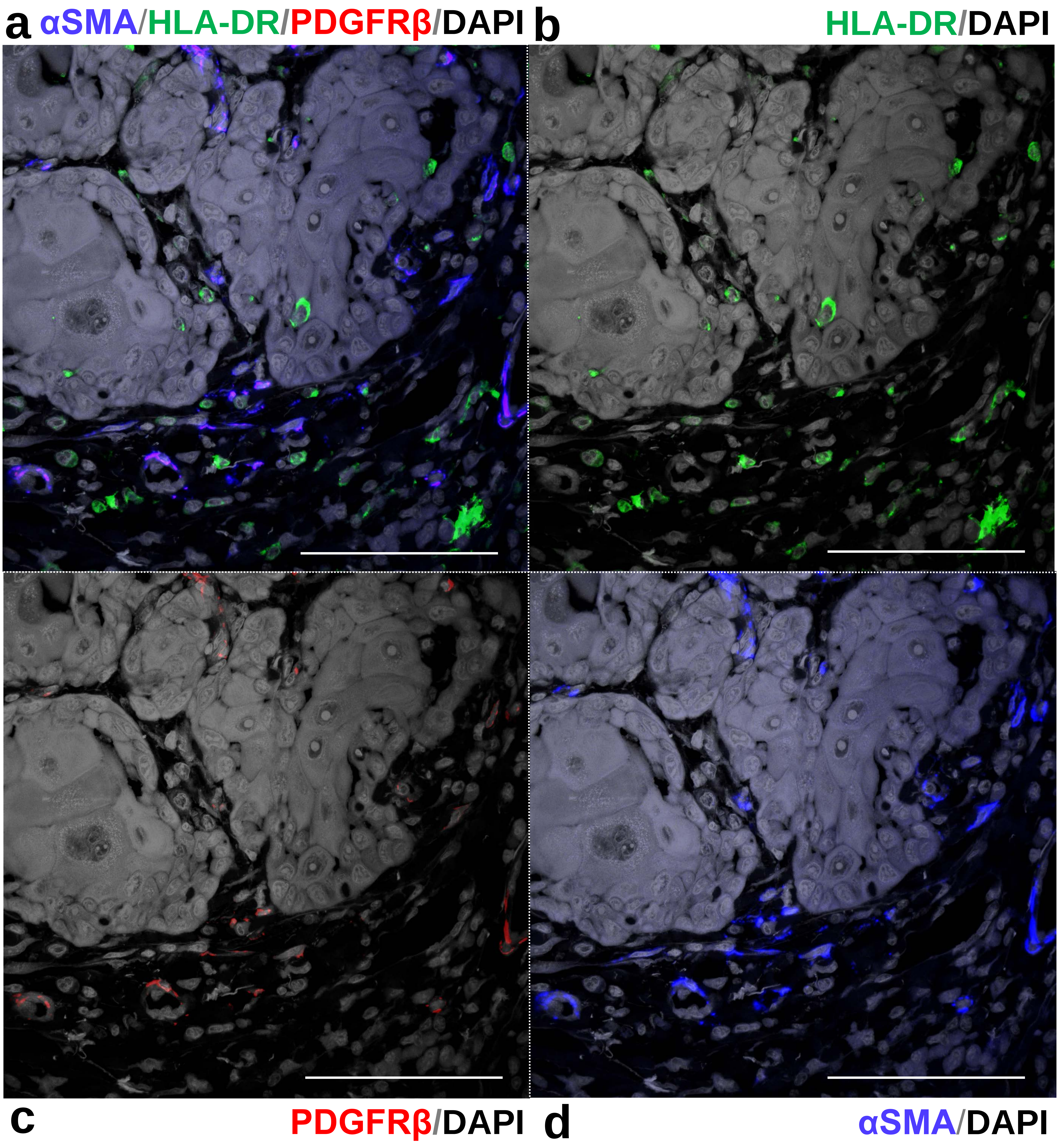

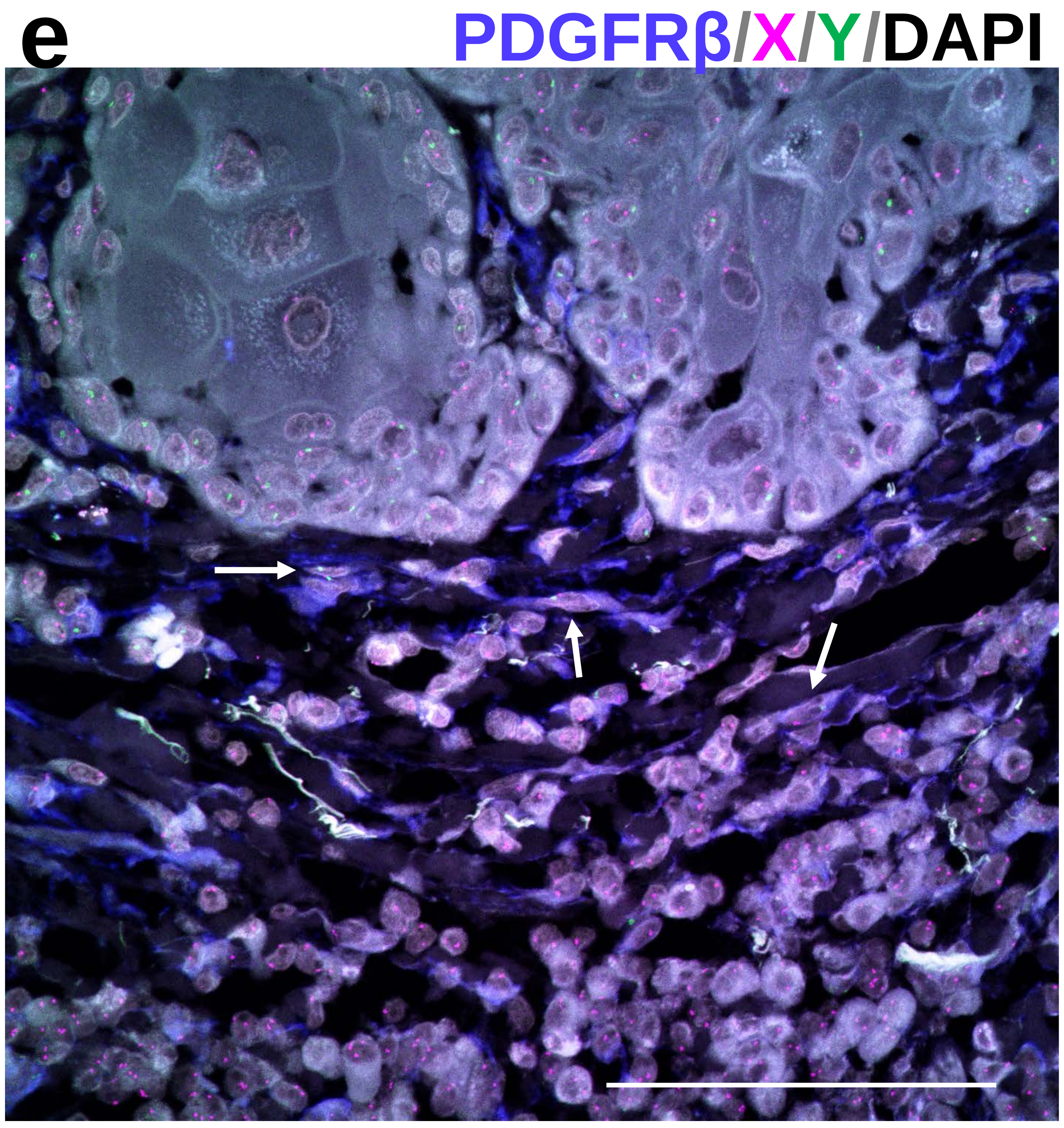

Supplementary Fig. 3 Triple immunostaining and immunoFISH for bone marrow stromal cell markers in case 3.

a–d, Triple immunostaining for αSMA, HLA-DR, and PDGFRβ. 97% of 115 αSMA(+) SCSSNs in 10 HPFs also expressed PDGFRβ. PDGFRβ(+) αSMA(-) SCSSNs and αSMA(+) HLA-DR(+) SCSSNs were sparse (< 1% of the PDGFRβ(+)SCSSNs, αSMA(+)SCSSNs in 10 HPFs, respectively; > 100 cells were analyzed). αSMA, blue; PDGFRβ, red; HLA-DR, green; DAPI, gray. Scale bar, 100 μm.

e, PDGFRβ-immunoFISH analysis in the tumor specimen. Recipient-derived PDGFRβ(+) cells are indicated by white arrows. The chimerism rates are shown in Fig. 5b. PDGFRβ, blue; X, magenta; Y, green; DAPI, gray. Scale bar, 100 μm.

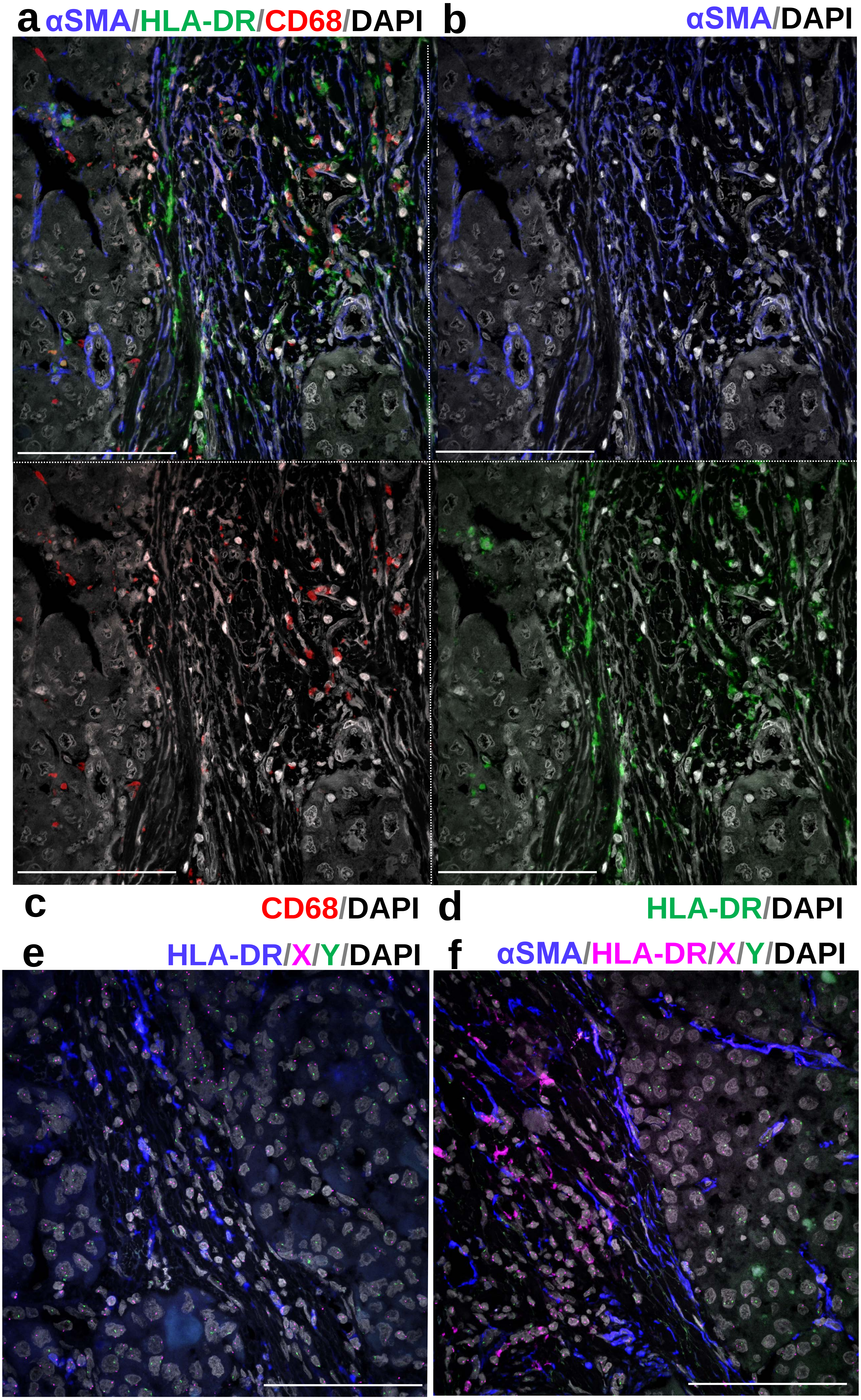

Supplementary Fig. 4. Triple immunostaining and immunoFISH with double immunostaining for macrophage and dendritic cell markers in case 2.
a–d, Triple immunostaining for αSMA, HLA-DR, and CD68. Scale bas,100 μm.
d 73% of 158 αSMA(-) SCSSNs were HLA-DR(+) and 72% of 116 HLA-DR(+) SCSSNs were CD68(+) cells. (in 10 HPFs). αSMA, blue; CD68, red; HLA-DR, green; DAPI, gray. Scale bar,100 μm.
e, HLA-DR-immunoFISH. Almost all HLA-DR(+) SCSSNs originated from the donor. The proportions of donor cells are shown in Fig. 7b. HLA-DR, blue; X, magenta; Y, green; DAPI, gray. Scale bar,100 μm.
f, ImmunoFISH for αSMA and HLA-DR. αSMA, blue; HLA-DR, magenta; X, magenta; Y, green; DAPI, gray. Scale bar, 100 μm.

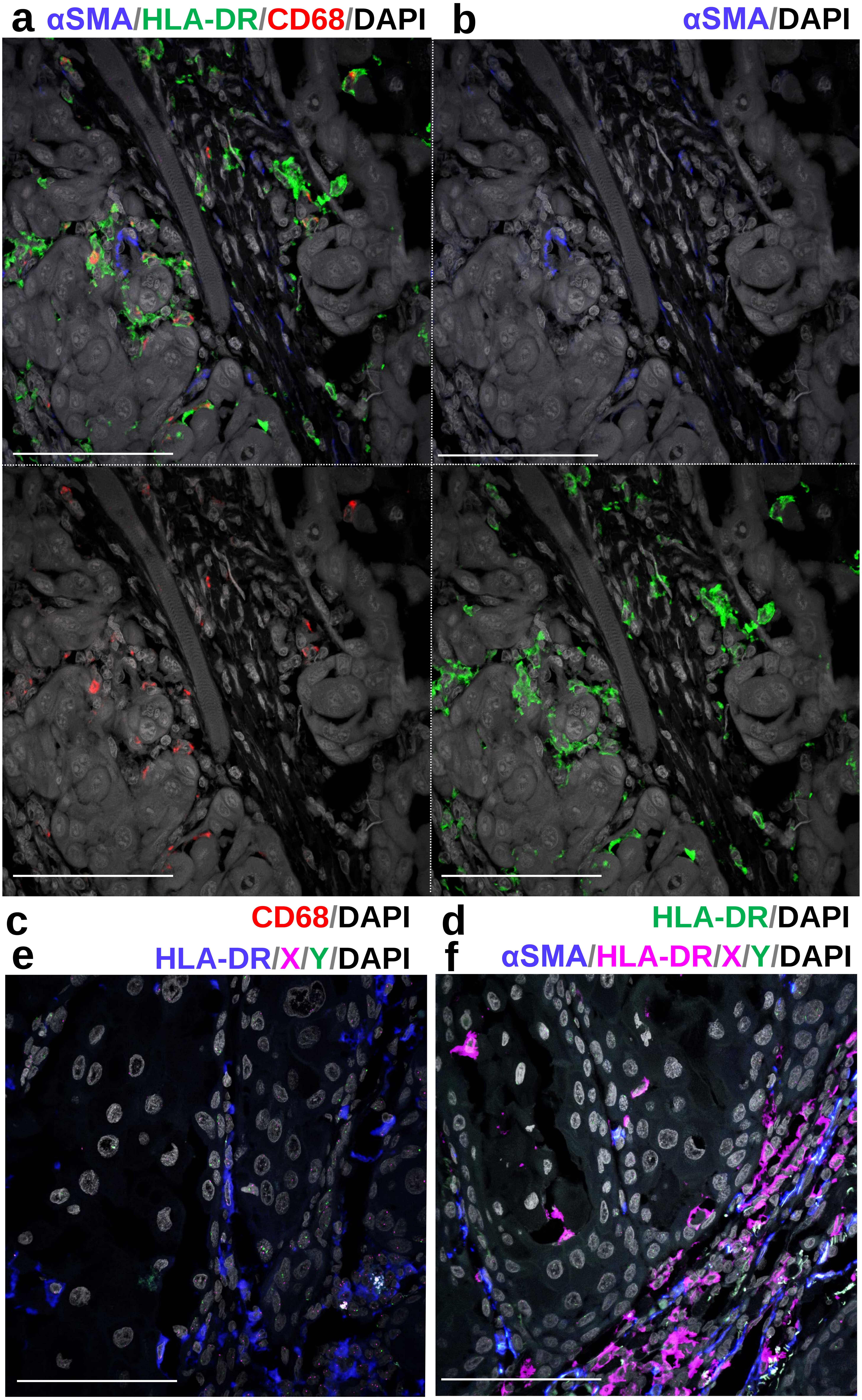

Supplementary Fig. 5. Triple immunostaining and immunoFISH with double immunostaining for macrophage and dendritic cell markers in case 3.

a–d, Triple immunostaining for αSMA, HLA-DR, and CD68. 76% of 141 αSMA(-) SCSSNs were HLA-DR(+) and 81% of 107 HLA-DR(+) SCSSNs were CD68(+) cells. (in 10 HPFs). αSMA, blue; CD68, red; HLA-DR, green; DAPI, gray. Scale bars, 100 μm.

e, HLA-DR-immunoFISH image. The proportions of donor cells are shown in Fig. 7b. HLA-DR, blue; X, magenta; Y, green; DAPI, gray. Scale bar, 100 μm.

f, ImmunoFISH for αSMA and HLA-DR. αSMA, blue; HLA-DR, magenta; X, magenta; Y, green; DAPI, gray. Scale bar, 100 μm.

Sample	Number of CD163(+) cells analyzed	Number of HPFs (sections) analyzed	Number of Bone marrow-derived CD163(+) cells	Bone marrow-derived CD163(+) cells (percent)
Case 1, breast carcinoma (tumor area)	321	76 (2)	321	100.0%
Case 1, Mammary gland tissue (non-tumor area)	302	148 (2)	302	100.0%
Case 2, Hepatocellular carcinoma (tumor area)	302	101 (3)	301	99.7%
Case 2, Liver tissue (non-tumor area)	412	136 (3)	410	99.5%
Case 3, Oral squamous cell carcinoma (tumor area)	324	36 (2)	324	100.0%
Case 3, Oral mucosa (non-tumor area)	301	65 (2)	301	100.0%

Supplementary Table 1. CD163-immunoFISH data in Cases 1–3.
